# Causal effect of lifestyle and metabolic indicator with herpes zoster: a two-sample Mendelian randomization study

**DOI:** 10.3389/fnut.2024.1433570

**Published:** 2024-08-14

**Authors:** Mingsheng Huang, Yiheng Liu, Cheng Chen, Weiran Dai

**Affiliations:** ^1^Department of Neurosurgery, The Second Affiliated Hospital of Chongqing Medical University, Chongqing, China; ^2^Department of Cardiology, The Second Affiliated Hospital of Chongqing Medical University, Chongqing, China; ^3^Department of Intensive Care Unit, JianYang Hospital of Traditional Chinese Medicine, Chengdu, China

**Keywords:** risk factors, metabolic indicator, herpes zoster, Mendelian randomization, causal effect

## Abstract

**Background:**

Previous observational studies have reported certain causal relationships between factors such as smoking, alcohol consumption, obesity, physical activity, metabolic disorders, and the incidence of herpes zoster (HZ). However, there is controversy regarding the observed results across different studies. Our objective was to investigate the causal effects of these risk factors on the risk of herpes zoster through a Mendelian randomization analysis using two-sample bidirectional approaches.

**Methods:**

We conducted two-sample bidirectional Mendelian randomization analyses to explore the causal relationships between different lifestyles, obesity assessment indices, metabolic indicators, and the risk of herpes zoster. All exposure and outcome data were sourced from publicly available data from genome-wide association studies.

**Results:**

In the inverse-variance weighted (IVW) analysis, body mass index (BMI) (OR: 1.160, 95% CI: 1.030–1.307, *p* = 0.014), Body fat percentage (BFP) (OR: 1.241, 95% CI: 1.050–1.467, *p* = 0.011), and whole body fat mass (WBFM) (OR: 1.199, 95% CI: 1.057–1.362, *p* = 0.005) exhibited positive associations with the risk of HZ. However, usual walking pace (UWP) (OR: 0.498, 95% CI: 0.254–0.976, *p* = 0.042) demonstrated a significant negative correlation with HZ risk. Other factors including alcohol intake frequency, smoking initiation, smoking status, insomnia, and sleep duration did not show significant causal relationships with HZ.

**Conclusion:**

Mendelian randomization studies revealed that BMI, BFP, and WBFM are risk factors for HZ. UWP showed a protective effect against HZ. These findings provide a straightforward method for evaluating future clinical practices aiming to develop personalized management strategies and assess high-risk populations for HZ.

## 1 Introduction

Herpes zoster (HZ), also known as shingles, originates from the same virus that causes chickenpox during childhood (1). In herpes zoster, the varicella-zoster virus (VZV) is reactivated, leading to the most common cause of painful rash. The reactivation of the VZV is a consequence of cell-mediated immune decline (2, 3). According to the Global Burden of Disease database, the mortality rate due to HZ in patients aged 65 and above ranges from 0.0022 to 82.21 per 100,000, indicating a substantial global health burden (4). Several factors are recognized as increasing the risk of HZ, including age, immunosuppression, infections, and psychological stress (5). Moreover, there is a correlation between certain modifiable factors and the risk of HZ. But research findings have been inconsistent. Some studies suggest that smoking might lower the risk of HZ (6–8), while others do not support this claim (9, 10). Similarly, while some research points to a correlation between alcohol consumption and the onset of HZ (11). However, some studies have indicated that alcohol consumption is not associated with HZ (9, 12). This inconsistency extends to other risk factors as well, such as obesity, metabolic changes, and intense physical activity. During the initiation of HZ, compromised immune function is conventionally acknowledged as the principal determinant. Some factors, including obesity (13), metabolic changes (14), and strenuous exercise (15) have been shown to exert certain immunosuppressive effects. However, some previous observational studies have not found an association between obesity or vigorous exercise and the incidence of HZ (6, 9, 16). Another study included 8,424 males and 10,634 females aged 50 years and older, stratified into three groups based on body mass index (BMI) cutoffs of 18.5 and 25.0 (underweight, normal weight, overweight). It was observed that the incidence rate of HZ in the overweight group was lower than that in the normal weight group among all participants and females (17).

To address these uncertainties, Mendelian Randomization (MR) provides a valuable methodological approach. MR uses genetic variability (single nucleotide polymorphisms, SNPs) as instrumental variables (IVs) to deduce causal links between exposures and diseases (18). Since genetic variations are randomly distributed before conception and remain stable, MR addresses limitations inherent in traditional observational studies, allowing for a more precise examination of causal relationships (19). Two-sample MR analysis enhances this by using summary statistics from genome-wide association studies (GWAS), increasing sample sizes and providing genetic insights (20). This research aims to employ MR to explore causal relationships between lifestyle factors, obesity indicators, metabolic changes, and HZ, with the objective of identifying risk factors and informing management strategies for HZ.

## 2 Methods

MR analysis is founded on three fundamental assumptions (19): (1) Instrumental variables (IVs) must be correlated with the exposure of interest; (2) IVs should be independent of confounding factors that might distort the causal relationship between the exposure and the outcome; (3) IVs must influence the outcome exclusively through their effect on the exposure, without any direct association with the outcome due to pleiotropy.

### 2.1 Data sources

We acquired GWAS from publicly accessible including lifestyle [alcohol intake frequency, smoking initiation, smoking status, Insomnia, sleep duration, moderate to vigorous physical activity, and usual walking pace (UWP)], metabolic biomarker [High-Density Lipoprotein cholesterol (HDL-C), Low-Density Lipoprotein cholesterol (LDL-C), total cholesterol (TC), triglycerides (TG), serum albumin, fasting glucose], obesity indicator [BMI, body fat percentage (BFP), whole body fat mass (WBFM), waist circumference (WC), hip circumference (HC), waist-hip ratio (WHR)], and metabolic disease [hypertension, type 1 diabetes mellitus (T1DM), type 2 diabetes mellitus (T2DM)]. The GWAS for herpes zoster were sourced from the FinnGen dataset, which includes 5,488 cases and 396,378 controls of Europe. The FinnGen dataset represents a substantive research initiative focused on elucidating the correlations between genetics and diseases, while also advancing the field of personalized medicine. By aggregating genetic data and health records from a multitude of individuals in Finland, researchers aim to identify genetic variations associated with a range of diseases and physiological traits. Ultimately, the goal is to enhance understanding of the genetic basis of diseases, thereby providing more accurate guidance for disease prevention, early detection, and treatment. This research endeavor holds significant implications for the advancement of genetic investigations, the promotion of health sciences, and the refinement of medical interventions (21). Further details about the dataset can be accessed at https://www.finngen.fi/en. These datasets are openly available for download and do not contain individual-level information, thereby eliminating the requirement for supplementary ethical review. The data sources are in Supplementary Table S1.

### 2.2 Genetic instrumental variable selection

In the forward MR analyses, the significance threshold was set at *p* < 5 × 10^−8^ to filter SNPs strongly associated with the exposure. Based on prior research experience, in the reverse MR analysis, due to the limited sample size of HZ, the available SNPs were restricted, and we selected a threshold of *p* < 5 × 10^−6^ to select available SNPs. Subsequently, we conducted a linkage disequilibrium clumping and excluded SNPs with *r*^2^ > 0.001 and a clump distance <10,000 kb. Then, we employed the *F*-statistic to assess the exposure correlations of instrumental variables. Calculating the *F*-value for each SNP allowed us to determine the presence of weak instrumental variable bias. A value of *F*-value >10 indicates a robust correlation, thus mitigating concerns of weak instrumental variable bias. The calculation of the *F*-value employs the formula *F* = *R*^2^/(1 − *R*^2^) × (*N* − 2) to determine the *F*-statistic for each SNP, where *R*^2^ represents the proportion of exposure variance explained by the instrumental variables, and *N* denotes the sample size. Additionally, we utilized the equation *R*^2^ = *β*^2^/(*β*^2^ + se^2^ × *N*) to evaluate the proportion of exposure variance explained by the instrumental variables, where *β* denotes the effect size of the genetic variant under investigation, she represents the standard error of *β*, and *N* signifies the sample size.

### 2.3 MR analyses

**Figure 1:**
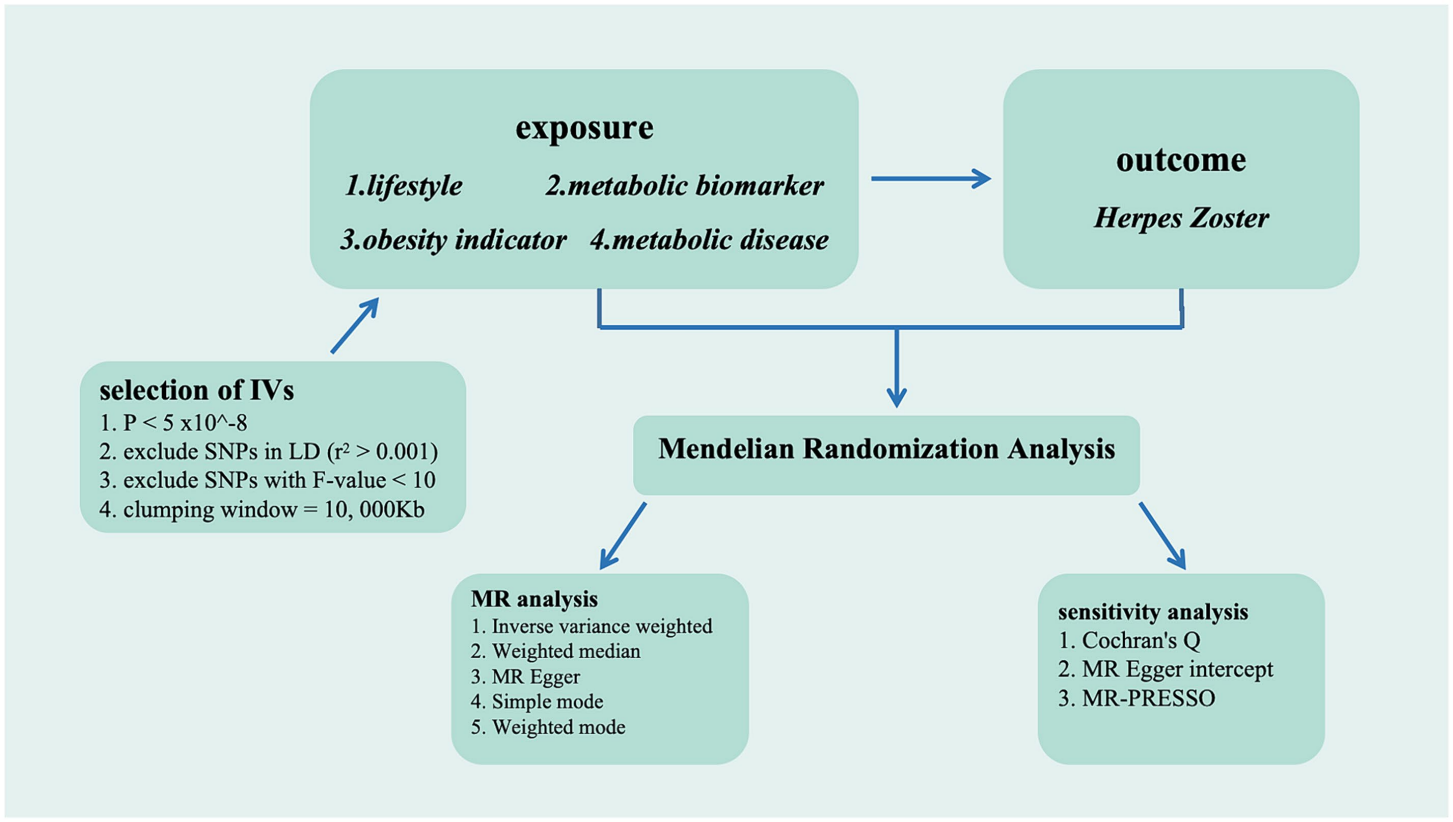
The flowchart depicting the Mendelian randomization procedure.

We utilized the inverse-variance weighted (IVW) method as the primary MR analysis within the random-effect model framework. This methodology integrates the Wald ratio of each chosen SNP through meta-analytical techniques to produce an estimated causal effect. The Wald ratio, calculated by dividing an SNP gene-outcome association by its gene-exposure association, serves as the SNP causal estimate. Although the IVW method offers the most precise estimation of causal effects, it is susceptible to the influence of invalid instruments with pleiotropic effects (22). To enhance the robustness of our findings, we therefore utilized four other distinct analytical approaches, including Weighted Median, MR Egger analysis, Simple mode, and Weighted Mode. In addition, we employed the MR-Egger intercept and examined multicollinearity. In sensitive analyses, Cochran’s *Q*-statistic assessed the heterogeneity among SNPs in the estimation obtained by the IVW method in this study, with a one-tailed *p*-value <0.05 considered significant heterogeneity within the *Q*-statistic. The MR-Egger intercept test was employed to evaluate the presence of horizontal pleiotropy in significant SNPs, with a one-tailed *p*-value <0.05 indicating horizontal pleiotropy. In addition, the MR-PRESSO method was employed to assess the presence of pleiotropy, where a one-tailed *p*-value <0.05 indicates statistical significance. Horizontal pleiotropy can be corrected by removing outliers, and substantial changes in the causal effect before and after outlier removal are evaluated. Furthermore, a combination of scatter plots and funnel plots was used to comprehensively evaluate the accuracy and reliability of the analytical outcomes. Sensitivity analysis was conducted by systematically excluding one SNP at a time to ascertain if any individual SNP independently influenced the causal relationship. Thus, employing the aforementioned methods aids in mitigating potential biases and confounding factors, thereby enhancing the accuracy of assessing the causal relationship between genetic variations and outcomes. The flowchart is available in Figure 1.

Estimates of the effect were presented as odds ratios (ORs) along with their respective 95% confidence intervals (CIs). Statistical significance was determined by two-tailed *p*-values <0.05. All computations were performed using the “TwoSampleMR” package (version 0.6.2) integrated into R software (version 4.3.3).

## 3 Results

According to the IV selection criteria, the effective SNPs for BMI, BFP, WBFM, and UWP in our primary outcome are 445, 376, 403, and 50, respectively. All *F*-values exceed 10, indicating no weak bias in the chosen instrumental variables. The summary table is available in Table 1, while the specific information about SNPs can be found in Supplementary Tables S2–S5.

**Table 1:** The results of SNP selection.

Trait	*P*	Number of SNPs	*R*2	Fmin
Body mass index	<5×10^−8^	445	5.25%	27.64
Body fat percentage	<5×10^−8^	376	4.84%	29.86
Whole body fat mass	<5×10^−8^	403	5.56%	29.75
Usual walking pace	<5×10^−8^	50	0.48%	29.79

**Figure 2:**
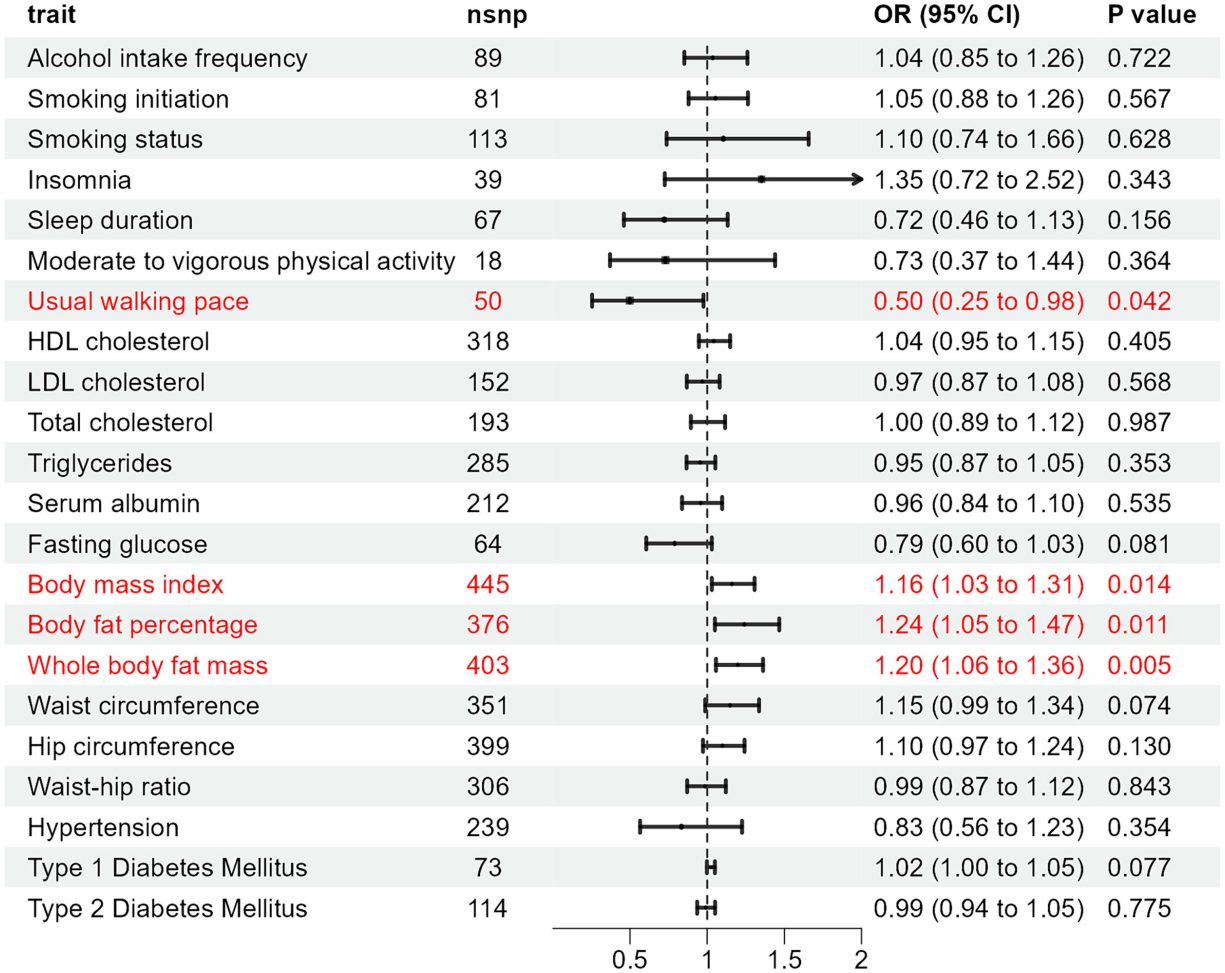
Causal effect of exposure to herpes zoster by IVW.

**Figure 3:**
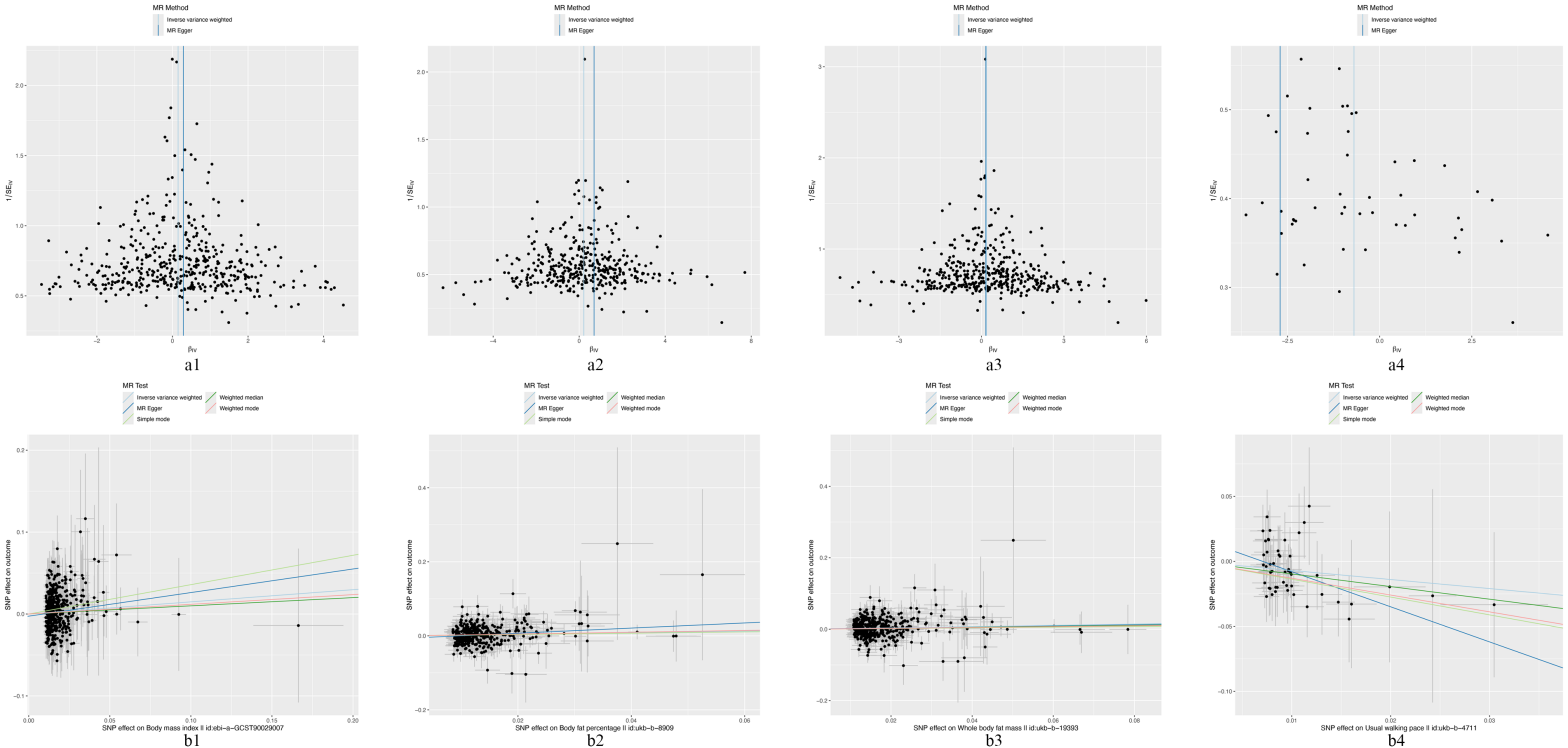
The funnel plots and scatter plots of BMI **(a1,b1)**, BFP **(a2,b2)**, WFPM **(a3,b3)**, and UWP **(a4,b4)** to herpes zoster.

In the MR analysis using the IVW method, three indicators showed significantly higher odds ratios (ORs) for herpes zoster: BMI (OR: 1.160, 95% CI: 1.030–1.307, *p* = 0.014), BFP (OR: 1.241, 95% CI: 1.050–1.467, *p* = 0.011), and WBFM (OR: 1.199, 95% CI: 1.057–1.362, *p* = 0.005). However, UWP exhibited a significant negative causal relationship with herpes zoster (OR: 0.498, 95% CI: 0.254–0.976, *p* = 0.042). No significant associations were found for genetic predictions of alcohol intake frequency, smoking initiation, smoking status, insomnia, sleep duration, moderate to vigorous physical activity levels, UWP, HDL-C, LDL-C, TC, TG, serum albumin levels, WC, HC, WHR, hypertension, T1DM, and T2DM (Figure 2 and Supplementary Table S6). This indicates that the selected SNPs serve as effective instrumental variables, accurately representing the exposure factors, thereby enhancing the reliability and robustness of the analytical outcomes. In our study, except for HDL-C, all *p*-values obtained from Cochran’s *Q* test for the exposures were above 0.05, indicating no heterogeneity. All MR-Egger intercepts clustered around zero, and all *p*-values >0.05, indicating no potential directional pleiotropy. The identification of a Global test *p*-value below 0.05 during MR-PRESSO analysis concerning HDL-C, persisting even after the removal of outliers, indicates the collective influence of various factors on HDL cholesterol levels. Consequently, ascertaining a causal relationship between HDL-C and the outcome becomes arduous (Supplementary Table S7). In addition, for exposures significantly associated with the risk of herpes zoster, both the scatter plot and funnel plot revealed no prominent outliers or biases (Figure 3). The forest plot showed that all effect estimates were consistently aligned without noticeable heterogeneity (Supplementary Figure S1). Furthermore, the leave-one-out analysis indicated that no individual SNP significantly influenced the outcomes (Supplementary Figure S2).

In the reverse MR analysis, 14 SNPs associated with HZ have been identified (Supplementary Table S8) according to the IV selection criteria. The results indicate no significant causal relationship between HZ and the three risks mentioned above factors and one protective factor: BMI (OR: 0.999, 95% CI: 0.977–1.021, *p* = 0.935), BFP (OR: 1.009, 95% CI: 0.993–1.024, *p* = 0.275), WBFM (OR: 1.011, 95% CI: 0.996–1.026, *p* = 0.152), and UWP (OR: 0.999, 95% CI: 0.991–1.008, *p* = 0.872). The detailed results are shown in Supplementary Table S9.

## 4 Discussion

MR is a valuable tool for assessing causal relationships in diseases. Recent studies have explored causal relationships between immune system-related factors and HZ. Zou et al. utilized MR to investigate the relationship between immune system-related factors and herpes zoster, offering deep insights into the role of immune function in the pathogenesis of herpes zoster (23). Additionally, Li et al. employed MR analysis to explore how immune function influences the risk of other diseases, including herpes zoster and its immune-related factors (24). These studies provide valuable insights into the potential causal links between immune function and HZ.

In our study, we employed Mendelian Randomization analysis to assess the causal relationships between seven lifestyle factors, six metabolic biomarkers, six obesity indicators, and three metabolic diseases with herpes zoster. The results revealed a positive association between genetically predicted BMI, BFP, and WBFM with the morbidity of HZ, whereas UWP showed a negative association with the risk of developing HZ.

BMI is a straightforward and effective tool for assessing the correlation between weight and height, offering a clear means of evaluating levels of obesity. Multiple studies suggest that there is no causal association between BMI and herpes zoster (6, 9, 12). One observational study stratified BMI into three groups and reported relative risk estimates for herpes zoster among overweight and obese individuals compared to those with normal or low body weight ranging from 1.00 to 1.04^6^. This result is consistent with our study findings. Our research investigated the relationship between BMI and HZ at the genetic level, revealing that it can impact the onset of HZ. During an acute herpes zoster outbreak, latent viruses become activated, replicate, and propagate along the affected nerves, ultimately eliciting an inflammatory immune response capable of damaging peripheral and central neurons (25). Obesity correlates with changes in subsets of lymphocytes in peripheral blood, heightened generation of inflammatory cytokines, raised levels of acute-phase proteins, and activation of inflammatory signaling cascades (26). Therefore, BMI may influence the occurrence of HZ through inflammatory pathways and potentially correlate with the severity of HZ. BFP denotes the ratio of fat tissue within an individual’s body mass. WBFM signifies the cumulative fat content across the entire physique, comprising both indispensable fat crucial for bodily functions and surplus fat stored beneath the skin and around organs. These parameters serve to gauge obesity and its severity. Unlike BMI, BFP offers a more direct reflection of an individual’s adiposity, thereby facilitating a more precise evaluation of obesity. WBFM provides a quantified measure of the total fat within the body. These three metrics assess obesity from varied perspectives and collectively anticipate elevated risks of HZ, thereby fortifying the reliability of our research findings.

UWP typically refers to the speed at which individuals walk in their regular daily activities. Individual walking speed can be considered an indicator for assessing their overall health condition. Prior research has shown a correlation between walking speed and overall mortality, especially in the elderly population, with self-reported walking speed potentially serving as a crucial marker for cardiovascular health (27). Research suggests that a slower pace of walking may be associated with health issues such as cardiovascular diseases, obesity, and tumor (27, 28). However, there is currently no evidence suggesting its association with the incidence of herpes zoster. Our research indicates a negative correlation between UWP and the incidence of HZ. Potential explanations for this association include the UWP serving as an indicator of general health, encompassing the functionality of the immune system. Generally, a brisker walking pace correlates with heightened immune function owing to the immunostimulatory effects of exercise on the generation and activity of immune cells, moderate exercise has multiple positive effects on the immune system, including enhancing the function and activity of immune cells, promoting the generation and maturation of immune cells, reducing chronic inflammation levels, boosting antibody production, facilitating the formation and maintenance of immune memory, and increasing the immune tolerance of the body (29, 30). These effects enable the body to more effectively combat pathogen invasion, enhance immune function, and thus improve resistance to diseases.

In addition, in the MR-PRESSO analysis, the global test *p*-value for HDL-C was <0.05 and remained significant even after the removal of outliers. This indicates a collective influence of multiple factors (such as genetics, lifestyle, health conditions, and medication use) on HDL cholesterol levels. This may suggest potential issues of multicollinearity within the model, making it challenging to determine the causal relationship between HDL-C and herpes zoster. Future research could employ different instrumental variable methods and larger datasets to more accurately estimate the causal effect of HDL-C on the outcome.

Our investigation revealed no significant correlation between blood lipid levels, serum albumin levels, and the risk of HZ, indicating a lack of influence of these factors on HZ susceptibility. Although some observational studies have suggested diabetes as a potential risk factor for heightened HZ susceptibility (31, 32), the current evidence remains inconclusive. Our study did not detect any associations between fasting blood glucose levels, type 1 diabetes, type 2 diabetes, and increased risk of HZ. Moreover, contentious risk factors such as smoking and alcohol consumption did not exhibit a substantial causal relationship with HZ incidence in our analysis.

### 4.1 Strengths and limitations

Our research employed Mendelian Randomization to explore the causal links between lifestyle factors, obesity indices, metabolic biomarkers, and herpes zoster incidence. This approach helps develop evidence-based lifestyle and management strategies for at-risk patients. Mendelian Randomization uses natural genetic variation to address confounding and reverse causation issues typical in observational studies, mimicking randomized controlled trials at lower costs and higher ethical standards. The data were sourced from publicly available databases, ensuring reproducibility and transparency. However, our study has some limitations.

Although MR methods are designed to mitigate common confounding and reverse causation issues in observational studies, they cannot completely eliminate the influence of potential confounders. The GWAS data utilized in our study were sourced from European populations; hence, the generalizability of the findings to other ethnic groups requires validation across diverse populations. Certain exposures, such as the UWP, are influenced by various factors including age, cardiopulmonary function, height, and body size. HDL-C levels are affected by genetic factors, lifestyle, health conditions, and medication use. The interplay among these factors can introduce considerable variability in HDL-C levels and UWP, thereby complicating the determination of its causal relationship with herpes zoster. Furthermore, the absence of multiple testing correction may increase the risk of false positives. This multiplicity of influencing factors further complicates the identification of the primary mediators of herpes zoster, making it challenging to isolate direct causal relationships. Additionally, our HZ cases were obtained from public databases with a relatively small sample size, which may affect the robustness of our results. Initially, we set the significance threshold for exposure-related SNPs at *p* < 5 × 10^−8^, but due to the limited number of effective SNPs, we broadened this to *p* < 5 × 10^−6^, potentially introducing some instability in the findings.

Future research should address these limitations by increasing sample sizes and incorporating diverse racial and geographic populations to validate the generalizability of our results. Furthermore, appropriate multiple testing corrections should be applied to reduce the risk of false positives and enhance result reliability. Further clinical intervention studies are needed to better understand the causal relationships between lifestyle factors, obesity, metabolic biomarkers, and herpes zoster, providing insights into underlying mechanisms and supporting the development of personalized interventions to improve patient outcomes.

### 4.2 Perspective

The findings have significant implications for public health strategies aimed at preventing herpes zoster. The positive association between BMI, BFP, and WBFM suggests that obesity management may be an important preventive measure. Additionally, the negative association with UWP indicates that promoting physical activity could be beneficial in reducing HZ risk. Based on our findings, the following public health recommendations can be made: Firstly, the implementation and promotion of programs focused on obesity management and prevention could help reduce the incidence of herpes zoster. Secondly, encouraging regular physical activity, which enhances walking pace and overall health, may lower the risk of developing herpes zoster. Finally, further research is needed to validate these findings and explore additional preventive measures, particularly among high-risk populations for herpes zoster.

## 5 Conclusion

In conclusion, this study demonstrates that BMI, BFP, and WBFM may represent potential risk factors for herpes zoster, while UWP could be considered a potential protective factor against HZ. These findings offer a straightforward evaluation method for future clinical practices aiming to develop personalized management strategies and assess high-risk populations for HZ.

## Data Availability

The original contributions presented in the study are included in the article/Supplementary material, further inquiries can be directed to the corresponding author.

## Supplementary material

SUPPLEMENTARY FIGURE S1 The forest plot of BMI **(a1)**, BFP **(a2)**, WFPM **(a3)**, and UWP **(a4)** to herpes zoster.

SUPPLEMENTARY FIGURE S2 The leave-one-out sensitivity analysis of BMI **(d1)**, BFP **(d2)**, WFPM **(d3)**, and UWP **(d4)** to herpes zoster.

## Glossary

HZ: Herpes zoster
VZV: varicella-zoster virus
BMI: body mass index
MR: Mendelian randomization
SNPs: single nucleotide polymorphisms
IVs: instrumental variables
GWAS: genome-wide association studies
UWP: usual walking pace
HDL-C: High-Density Lipoprotein cholesterol
LDL-C: Low-Density Lipoprotein cholesterol
TC: total cholesterol
TG: triglycerides
BFP: body fat percentage
WBFM: whole body fat mass
WC: waist circumference
HC: hip circumference
WHR: waist-hip ratio
T1DM: type 1 diabetes mellitus
T2DM: type 2 diabetes mellitus
IVW: inverse-variance weighted
ORs: odds ratios
CIs: confidence intervals
