# Supplementary figures and images for: Causal effect of lifestyle and metabolic indicator with herpes zoster: a two-sample Mendelian randomization study
Source: Front Nutr. 2024 Aug 14;11:1433570. doi: 10.3389/fnut.2024.1433570 (PMC11351565; doi:10.3389/fnut.2024.1433570)

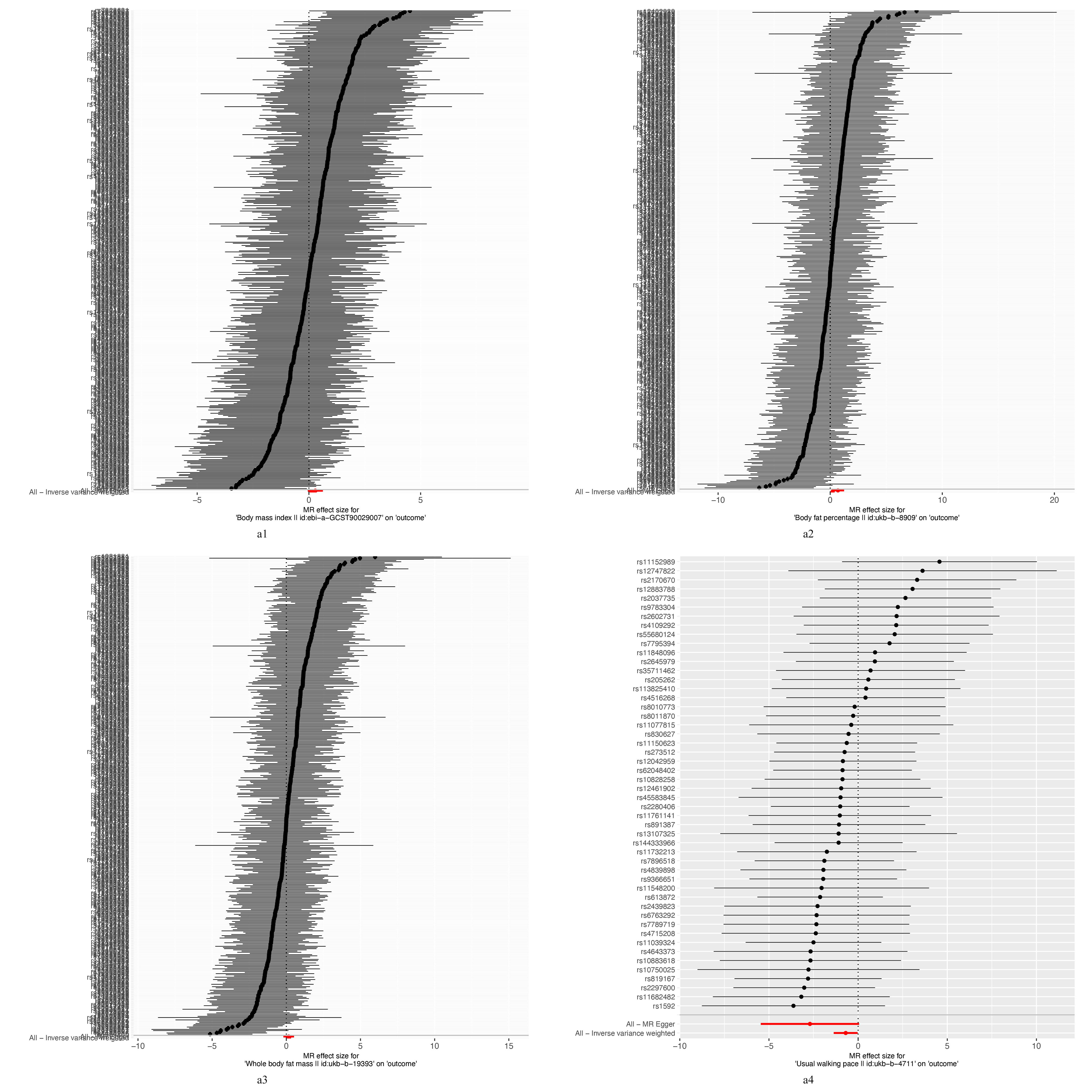

Supplement: SUPPLEMENTARY FIGURE S1 — The forest plot of BMI (a1), BFP (a2), WFPM (a3), and UWP (a4) to herpes zoster. [file Image_1.TIF]

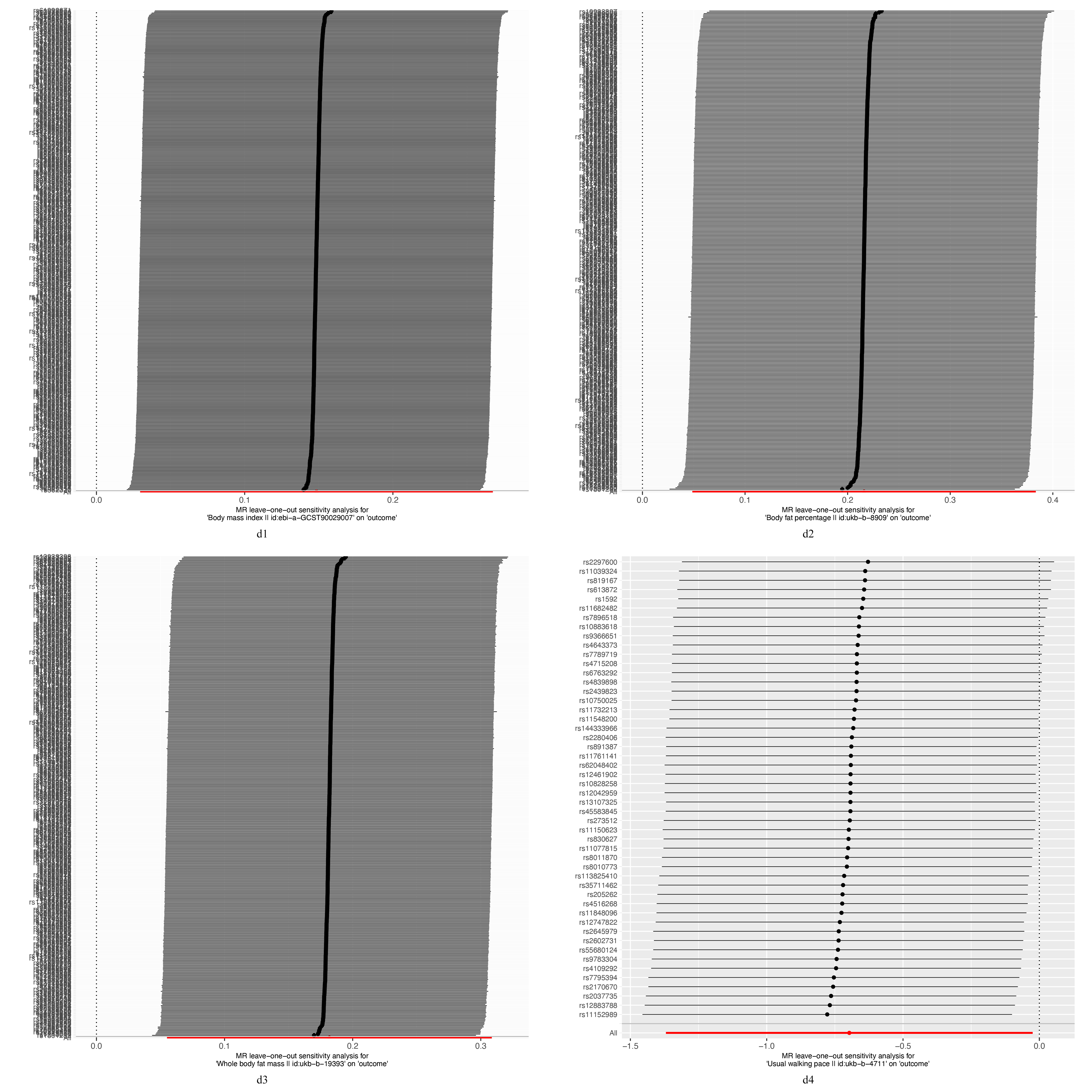

Supplement: SUPPLEMENTARY FIGURE S2 — The leave-one-out sensitivity analysis of BMI (d1), BFP (d2), WFPM (d3), and UWP (d4) to herpes zoster. [file Image_2.TIF]
